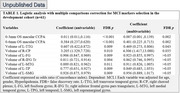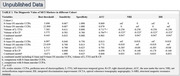# Perfusion Area of Macular Choriocapillaris: A Potential Biomarker of Mild Cognitive Impairment in Cerebral Small Vessel Disease Patients

**DOI:** 10.1002/alz70856_101949

**Published:** 2025-12-25

**Authors:** Weitao Yu, Sheng Zhang

**Affiliations:** ^1^ Hangzhou Normal University, Hangzhou, Zhejiang, China; ^2^ Zhejiang Provincial People's Hospital, Hangzhou, Zhejiang, China

## Abstract

**Background:**

To develop and validate markers for screening mild cognitive impairment (MCI) in cerebral small vessel disease (CSVD) using Swept Source Optical Coherence Tomography Angiography (SS‐OCTA).

**Method:**

Participants with MCI and normal cognition (NC) underwent structural magnetic resonance imaging (S‐MRI) and SS‐OCTA were prospectively recruited (Dream‐10 and FRESH‐CSVD study, NCT 06164262 and NCT06431711). Patients with Alzheimer's disease (AD) were excluded according to plasma biomarkers test. MCI was defined as a Montreal Cognitive Assessment (MoCA) score ranging from 18 to 26 points, accompanied by a complaint of memory loss. Participants were categorized into development (January 2024 to May 2024) and validation cohorts (June 2024 to September 2024) based on chronological order. LASSO‐derived logistic regression analysis was employed to filter potential markers, which was further validated via temporal validation.

**Result:**

A total of 102 participants were enrolled, with 59.8% (61/102) having MCI. In the development cohort (*n* = 61), the volume of left transverse temporal gyrus (L‐TTG), the volume of right choroid plexus (R‐CP) on S‐MRI, 0‐3mm and 0‐6mm oculus Sinister (OS) macular perfusion area (PA) of choriocapillaris (CC) were identified as MCI markers (FDR *p* < 0.05) (Table 1). In the validation cohort (*n* = 41), 0‐3mm and 0‐6mm OS macular CCPA were identified as superior MCI markers (AUC:0.849 and 0.833; sensitivity:0.672 and 0.639; specificity:0.976 and 0.951) with significant better NRI and IDI when compared to other MCI markers (all *p* < 0.05) (Table 2).

**Conclusion:**

SS‐OCTA, especially OS macular CCPA, holds promise for screening MCI in CSVD patients.